# Pulmonary Nodules and Splenic Lesions as Rare Extraintestinal Manifestations of Crohn’s Disease

**DOI:** 10.7759/cureus.105605

**Published:** 2026-03-21

**Authors:** Shane V Varghese, Julie N Nguyen, Shakeela F Shah, Jose M Garcia

**Affiliations:** 1 Family Medicine, Kaiser Permanente Woodland Hills Medical Center, Los Angeles, USA; 2 Internal Medicine, Kaiser Permanente Woodland Hills Medical Center, Los Angeles, USA

**Keywords:** corticosteroid responsiveness, crohn’s disease (cd), extra-intestinal manifestation of inflammatory bowel disease, pulmonary nodules, splenic lesions

## Abstract

Extraintestinal manifestations of Crohn’s disease are rare but should be considered in patients with newly discovered lesions in organs not typically affected by inflammatory bowel disease (IBD). Here, we describe a case of a patient who presented with abdominal pain due to splenic lesions, with incidental discovery of pulmonary nodules. The patient was also found to have methicillin-sensitive *Staphylococcus aureus* (MSSA) bacteremia on presentation, raising initial concern for septic emboli to the lungs and spleen. However, the patient’s abdominal pain did not improve, and the splenic lesions and pulmonary nodules increased in size despite appropriate antibiotic therapy, prompting evaluation for an alternative diagnosis. After further workup and multidisciplinary discussion, the patient was started on corticosteroids for suspected extraintestinal Crohn’s disease, with rapid clinical improvement. This case highlights the importance of reconsidering the differential diagnosis when a presumed etiology and its treatment do not explain the clinical course, and it reviews the literature on pulmonary nodules and splenic lesions as rare extraintestinal manifestations of Crohn’s disease.

## Introduction

Crohn's disease is a form of inflammatory bowel disease (IBD) characterized by skip lesions and transmural inflammation, which can affect any part of the gastrointestinal (GI) tract from the mouth to the anus. Symptoms usually appear in the late teens to twenties and include diarrhea, abdominal pain, fatigue, weight loss, fever, and rectal bleeding [1].

Crohn's disease can also have extraintestinal manifestations, the most common of which include joint conditions (axial and peripheral arthropathy), eye disorders (uveitis, scleritis, and episcleritis), skin conditions (pyoderma gangrenosum and erythema nodosum), and hepatobiliary disorders (primary sclerosing cholangitis). Other extraintestinal manifestations include thromboembolic disease, metabolic bone disease, and nephrolithiasis. Some immune-mediated processes have also shown an association with Crohn’s disease, including asthma, chronic bronchitis, psoriasis, rheumatoid arthritis, celiac disease, multiple sclerosis, and pericarditis [2].

Necrobiotic pulmonary nodules are a rare but increasingly recognized extraintestinal pulmonary manifestation of Crohn’s disease. Although only a handful of cases had been reported by 2009 [3-6], additional case reports have since expanded the published literature [7-14]. This case report presents a 2024 case of necrobiotic pulmonary nodules and splenic lesions in a Crohn’s disease patient. To our knowledge, these two findings have not been reported together previously.

## Case presentation

A 33-year-old male with a past medical history of Crohn’s disease diagnosed about 20 years prior and no other relevant conditions presented to the emergency department with left-sided abdominal pain for three weeks that radiated into the left lower back. Medications used to treat his Crohn’s disease included mesalamine 2.4 g daily and infliximab 400 mg every five weeks. A colonoscopy two months prior to presentation showed that his Crohn’s disease was in remission.

On examination, the patient was noted to have left upper quadrant and left lower quadrant abdominal tenderness to palpation. He also had chronic plaques all over his body without any signs of infection, secondary to a long-standing history of psoriasis. The rest of the physical examination was unremarkable. Initial laboratory results included a mildly elevated white blood cell count with neutrophil predominance, normal lipase, normal liver function tests, and elevated inflammatory markers (Table 1). Urinalysis showed pyuria and positive leukocyte esterase, but urine culture had less than 10,000 CFU/mL of insignificant growth.

**Table 1:** Laboratory data on presentation AST: Aspartate aminotransferase; ALT: Alanine aminotransferase.

Laboratory Value	Patient Value	Reference Range and Unit of Measurement
White blood cell count	11.3	4.0–11.0 x 1000/mcL
Hemoglobin	14.2	13.5–17.5 g/dL
Platelets	335	130–400 x 1000/mcL
Sodium	136	135–145 mEq/L
Potassium	3.3	3.5–5.0 mEq/L
Blood urea nitrogen	6	≤18 mg/dL
Creatinine	0.75	≤1.30 mg/dL
Glucose	96	70–140 mg/dL
Total bilirubin	0.6	≤1.0 mg/dL
Alkaline phosphatase	76	≤125 U/L
AST	21	≤34 U/L
ALT	16	≤63 U/L
Lipase	29	≤58 U/L
Erythrocyte sedimentation rate	57	0–15 mm/h
C-reactive protein	159.3	≤7.4 mg/L

**Figure 1:**
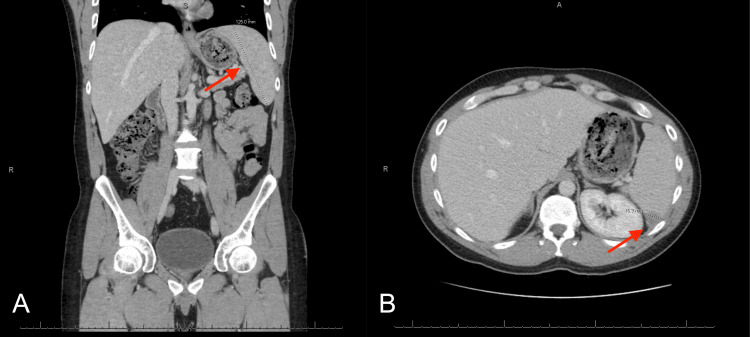
CT abdomen and pelvis with IV contrast (A) Splenomegaly measuring 12.5 cm. (B) Lesion in the posterior aspect of the spleen measuring up to 16.9 mm.

Computed tomography (CT) of the abdomen and pelvis with intravenous (IV) contrast showed mild splenomegaly measuring 12.5 cm and multiple hypodensities present throughout the spleen, with the largest measuring up to 16.9 mm in the posterior aspect (Figure 1).

**Figure 2:**
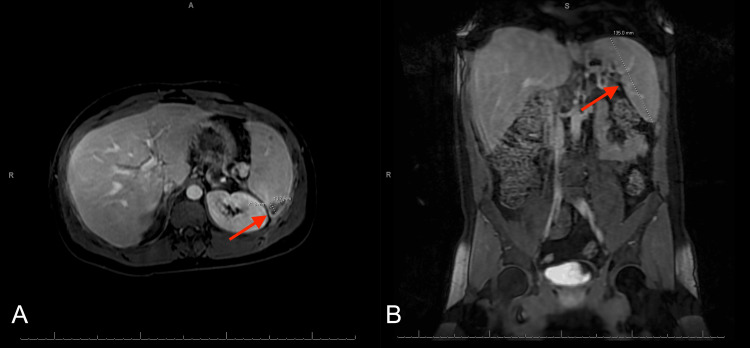
MRI abdomen and pelvis with and without IV contrast (A) Hypointense lesion in the posterior aspect of the spleen measuring approximately 2.1 x 1.3 cm. (B) Splenomegaly measuring 13.5 cm.

MRI abdomen and pelvis with and without IV contrast revealed a hypointense nonenhancing triangular area in the spleen posteriorly measuring approximately 2.1 x 1.3 cm, additional smaller irregularly shaped splenic hypoenhancing and hypodense lesions, and splenomegaly measuring 13.5 cm (Figure 2).

**Figure 3:**
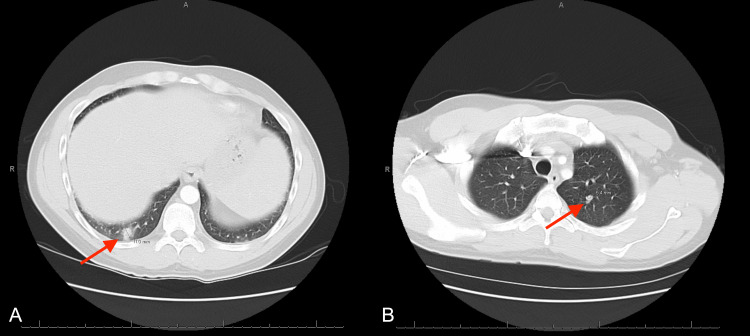
CT pulmonary angiogram (A) Lung nodule in the right lower lobe measuring 11 mm. (B) Lung nodule in the left upper lobe measuring 5.4 mm.

CT pulmonary angiogram with contrast showed multiple bilateral pulmonary nodules, with the largest measuring up to 11 mm in the right lower lobe (Figure 3).

**Figure 4:**
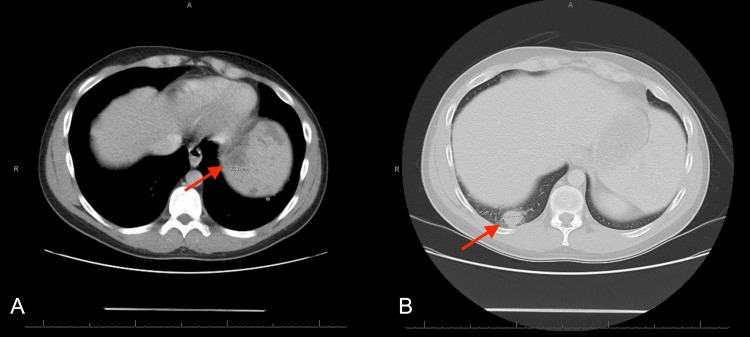
CT chest, abdomen, and pelvis with IV contrast (A) Multiple heterogeneous low attenuation lesions identified throughout the spleen, measuring up to 3 cm, significantly increased from prior CT. (B) Lung nodule in the right lower lobe measuring up to 2.5 cm increased from prior CT.

The patient was found to have positive blood cultures for methicillin-sensitive *Staphylococcus aureus* (MSSA) in one out of two sets and was started on treatment for suspected septic emboli to the lungs and spleen. The chronic plaques all over his body from his psoriasis were thought to be a potential source. After almost two weeks of IV oxacillin for treatment of MSSA bacteremia with no improvement in abdominal pain despite subsequent negative blood cultures, a repeat CT chest, abdomen, and pelvis with IV contrast was ordered to assess for interval changes, which showed interval worsening of the pulmonary nodules and splenic lesions (Figure 4).

**Figure 5:**
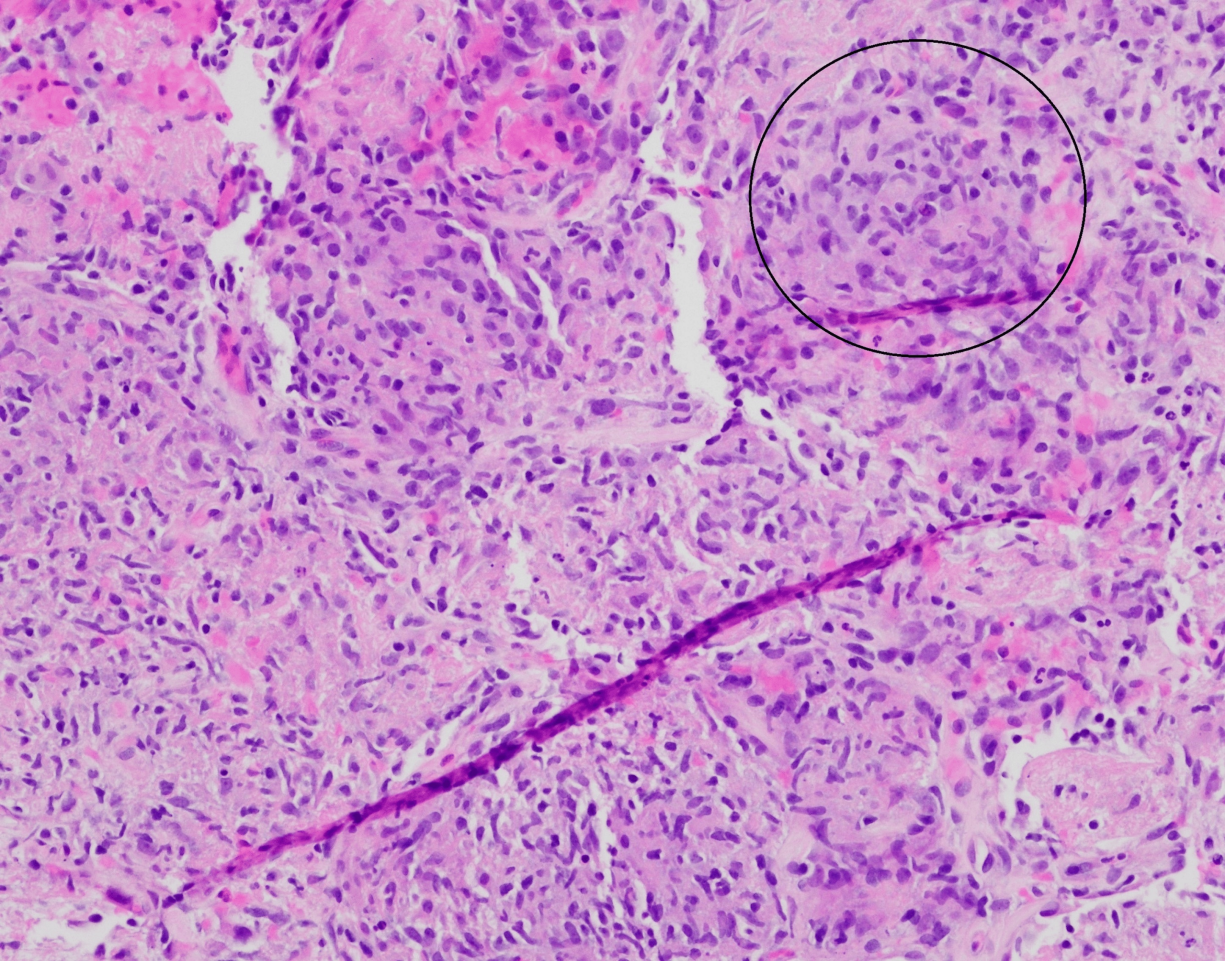
Mixed inflammation with vague granulomatous inflammation The black circle outline indicates granulomatous inflammation. Stain: Hematoxylin and eosin (H&E).

**Figure 6:**
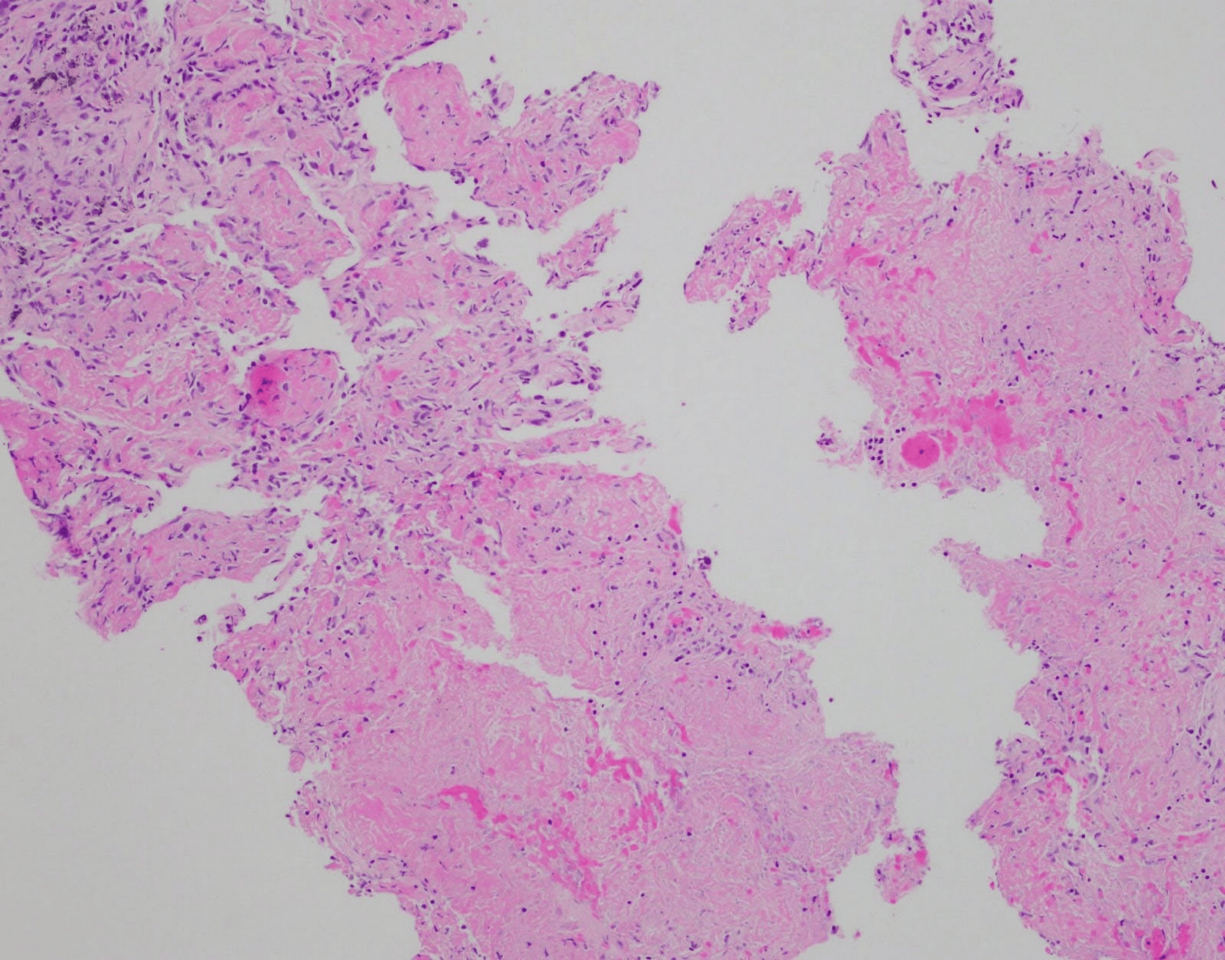
Fibrinous necrosis Stain: Hematoxylin and eosin (H&E).

CT-guided biopsy of a lung nodule revealed organizing acute lung injury with prominent airspace fibrin, necrosis, and vague non-necrotizing granulomatous inflammation (Figures 5, 6). Gram stain, fluorescent stains for acid-fast bacilli, and Warthin-Starry stain were all negative.

Initially, the differential posed by the pathologist included organizing acute lung injury secondary to acute infection, adverse drug reaction, septicemia, and systemic connective tissue disease. However, after multidisciplinary review, the clinical course and histopathology, including noncaseating granulomas as a hallmark feature of Crohn’s disease, were deemed most consistent with necrobiotic nodules associated with Crohn’s disease, a rare extraintestinal manifestation.

**Figure 7:**
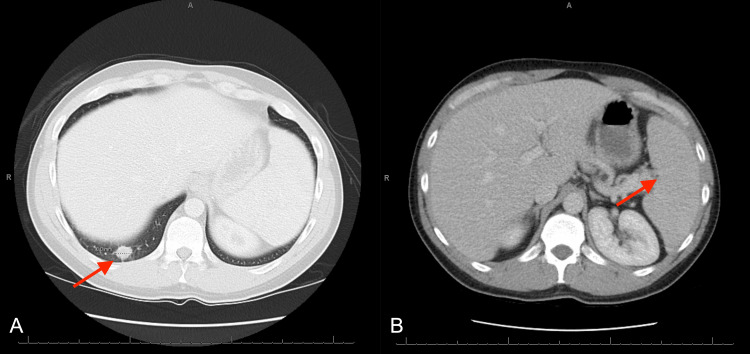
CT chest and abdomen with IV contrast (A) Lung nodule in the right lower lobe measuring 1.6 cm decreased from prior CT. (B) Decrease in diffuse splenic parenchymal heterogeneity compared to prior CT.

After four days of steroids, CT chest and abdomen with IV contrast showed decreased size of pulmonary nodules along with decreased number and size of splenic lesions (Figure 7). He was discharged with a one-month course of oral steroids before eventually restarting infliximab for his Crohn’s disease. His abdominal pain improved, and he was able to wean off pain medications. At a pulmonology follow-up appointment, repeat CT imaging was ordered, but he did not complete the study.

## Discussion

Crohn’s disease and its extraintestinal pulmonary manifestations

The disease course of Crohn’s disease varies for each patient. Many patients have intermittent and chronic symptoms. Some can have continual, progressive active disease. Approximately 20% of patients achieve prolonged remission [15]. Inflammation in Crohn’s disease is thought to be caused by immune dysregulation, particularly involving defective macrophage cytokine secretion and reduced neutrophil recruitment. The mucosal immune system mounts an insufficient acute response to luminal bacteria that breach the mucosa, impairing bacterial clearance and leading to secondary chronic inflammation [16].

The pathogenesis of pulmonary disease associated with IBD is not well understood. The type of inflammatory changes in the lungs is thought to be similar to those that occur in the bowel [17]. Furthermore, the respiratory and gastrointestinal tracts share an embryologic origin in the primitive foregut, which could theoretically predispose the lung to inflammatory changes in patients with Crohn’s disease [18].

Pulmonary manifestations in Crohn’s disease are less common than those in ulcerative colitis. Patterns of lung involvement in Crohn's disease include large and small airway disease, such as bronchitis, tracheobronchial stenosis, bronchiectasis, and bronchiolitis obliterans, as well as parenchymal processes, such as interstitial lung disease and sterile necrobiotic nodules [19]. Pulmonary involvement of Crohn’s disease can present with respiratory symptoms, such as dyspnea or cough. However, there are cases, such as the current one, where the patient presents without any of these symptoms. The initial presentation of our patient included abdominal pain attributed to the splenic lesions; pulmonary involvement was discovered incidentally during further workup.

Necrobiotic lung nodules and splenic lesions in Crohn’s disease

As previously mentioned, necrobiotic pulmonary nodules represent an exceptionally rare extraintestinal manifestation of Crohn's disease. Histologically, these nodules consist of sterile aggregates of inflammatory cells with central necrosis, often surrounded by vague non-necrotizing granulomatous inflammation [6,19]. They have also been described in ulcerative colitis and in other non-IBD related disease processes, such as rheumatoid arthritis [20]. When associated with Crohn's, the nodules usually form in patients with clinically active disease. However, similar to our case, Sanjeevi and Roy described a case of necrobiotic pulmonary nodules in a patient whose Crohn's disease was in remission at the time [3].

The diagnosis is made via biopsy and requires rigorous exclusion of infectious etiologies, as the treatment for these nodules usually requires steroids and immunomodulatory agents. In our case, exclusion of an infectious etiology was achieved by noting the increasing size of the lung nodules despite appropriate IV antibiotic therapy. Gram, acid-fast, and Warthin-Starry stains were also performed and came back negative. These necrobiotic pulmonary nodules usually have a favorable response to corticosteroids [6,19].

Splenic lesions are an exceedingly rare extraintestinal manifestation of Crohn’s disease. In general, splenic lesions warrant a broad differential diagnosis, including infection (bacterial, mycobacterial, and fungal), malignancy (leukemia and lymphoma), and systemic autoimmune/inflammatory conditions (systemic lupus erythematosus, rheumatoid arthritis, sarcoidosis, and common variable immunodeficiency) [21,22]. When alternative etiologies have been reasonably excluded, Crohn’s-associated splenic manifestations should be considered, most notably sterile splenic abscesses and granulomatous or rheumatoid-like lesions.

Sterile splenic abscesses have been reported in Crohn’s disease, often presenting with nonspecific symptoms and an otherwise negative infectious evaluation. Imaging may demonstrate hypodense nodules or multiloculated splenic lesions [21,23]. When tissue is available, pathology may show macrophages and histiocytes with a central area of necrosis containing damaged neutrophils, with no pathogenic microorganisms identified [23].

Noncaseating granulomas or rheumatoid-like nodules involving the spleen have also been described in Crohn’s disease. Reported cases include granulomatous lesions of the liver and spleen with prompt corticosteroid response [24] and rheumatoid-like granulomas that improved with immunosuppression but ultimately required splenectomy [25]. A key characteristic of these splenic manifestations of Crohn’s disease is their lack of response to antibiotics and a marked improvement with corticosteroids [21,23-25].

Although our patient’s splenic lesions were not biopsied, it is reasonable to infer that these lesions were of an aseptic inflammatory origin, given their marked, rapid response to steroids. Their cooccurrence with necrobiotic pulmonary nodules, which also responded favorably to steroids, also supports that these splenic lesions were of an aseptic inflammatory origin. Improvement, and sometimes resolution, of these lesions occurs with glucocorticoids. However, relapses may occur, and approximately half of patients may require additional immunosuppressive therapy [23].

## Conclusions

In patients with Crohn’s disease, clinicians should maintain suspicion for extraintestinal manifestations when respiratory and/or abdominal symptoms are unexplained or fail to improve with initial therapy. A thorough evaluation is essential to exclude alternative etiologies, including infection, malignancy, and autoimmune conditions. Once these have been reasonably excluded, early corticosteroid therapy should be considered, as rapid clinical and radiographic response can support a sterile inflammatory process and may hasten symptom resolution. Close follow-up is warranted to monitor for relapse and to determine whether additional immunosuppressive therapy is needed.
